# Adherence to Dietary Recommendations, Nutrient Intake Adequacy and Diet Quality among Pediatric Cystic Fibrosis Patients: Results from the GreeCF Study

**DOI:** 10.3390/nu12103126

**Published:** 2020-10-13

**Authors:** Dimitrios Poulimeneas, Maria G. Grammatikopoulou, Panagiota Devetzi, Argyri Petrocheilou, Athanasios G. Kaditis, Theodora Papamitsou, Stavros E. Doudounakis, Tonia Vassilakou

**Affiliations:** 1 Department of Public Health Policy, School of Public Health, University of West Attica, 196, Alexandras Avenue, GR-11521 Athens, Greece; dpoul@hua.gr; 2 Department of Nutrition and Dietetics, Harokopio University, E. Venizelou 70, GR-17671 Athens, Greece; 3 Department of Nutritional Sciences & Dietetics, Faculty of Health Sciences, International Hellenic University, Alexander Campus, GR-57001 Thessaloniki, Greece; maria@ihu.gr (M.G.G.); panagiwtadv@gmail.com (P.D.); 4 Faculty of Biotechnology, Universidade Católica Portuguesa, Rua de Diogo Botelho, 1327, 4169-005 Porto, Portugal; 5 Cystic Fibrosis Department, Agia Sophia Children’s Hospital, Thivon 1, GR-11527 Athens, Greece; apetroch@gmail.com (A.P.); kaditia@hotmail.com (A.G.K.); stavrosdoudounakis@yahoo.com (S.E.D.); 6 Division of Pediatric Pulmonology and Sleep Disorders Laboratory, First Department of Pediatrics, National and Kapodistrian University of Athens School of Medicine and Aghia Sophia Children’s Hospital, Thivon 1, GR-11527 Athens, Greece; 7 Laboratory of Histology and Embryology, Medical School, Faculty of Health Sciences, Aristotle University of Thessaloniki, University Campus, GR-54124 Thessaloniki, Greece

**Keywords:** Mediterranean diet, dietary recommendations, nutrition, medical nutrition therapy, growth, malnutrition, pediatric patients, nutritional assessment, pulmonological disease, nutritional status, lung disease

## Abstract

Nutrition is an important component of cystic fibrosis (CF) therapy, with a high-fat diet being the cornerstone of treatment. However, adherence to the dietary recommendations for CF appears suboptimal and burdensome for most children and adolescents with CF, leading to malnutrition, inadequate growth, compromised lung function and increased risk for respiratory infections. A cross-sectional approach was deployed to examine the degree of adherence to the nutrition recommendations and diet quality among children with CF. A total of 76 children were recruited from Aghia Sophia’s Children Hospital, in Athens, Greece. In their majority, participants attained their ideal body weight, met the recommendations for energy and fat intake, exceeding the goal for saturated fatty acids consumption. Carbohydrate and fiber intake were suboptimal and most participants exhibited low or mediocre adherence to the Mediterranean diet prototype. It appears that despite the optimal adherence to the energy and fat recommendations, there is still room for improvement concerning diet quality and fiber intake.

## 1. Introduction

Cystic fibrosis (CF) is a genetic disease, affecting primarily the respiratory and gastrointestinal systems. Treatment includes daily medication, enzymes, adherence to a high-fat diet, vitamin oral nutrient supplements (ONS), as well as physiotherapy and respiratory exercises. Treatment adherence is important to maintain health and prolong life expectancy, however, compliance to the therapy appears to be problematic, especially as far as diet, exercise and chest physiotherapy are concerned [1]. According to research, a great proportion of CF patients fail to comply to the general, pancreatic enzyme replacement therapy (PERT), vitamin prescription and respiratory exercises due to lack of motivation, time and interest [2,3,4], whereas 11% of patients homozygous for the *F508del*-Cystic Fibrosis Transmembrane conductance Regulator (CFTR) mutation do not adhere to the Lumacaftor-ivacaftor medication prescription [5]. In particular, persistent adherence appears to be the most problematic one, with very few patients exhibiting periods of complete adherence at some point in their life [6].

As far as adolescent patients are concerned, most do not comply to the daily vest therapy [7] and chest physiotherapy [8], and more than half are considered as low PERT adherers [8,9]. Nutrition-wise, research appears unanimous on the fact that more than half of the children with CF demonstrate suboptimal compliance to the prescribed vitamins supplementation [8,10]. Low adherence to the dietary recommendations in particular, may result in malnutrition, with immediate effects on the immune system and lung function, and an increased risk for infections and hospitalization [11,12,13]. Due to the unique psychological characteristics of adolescents with chronic disease and their frequent failure to conform to therapy, compliance surveillance is important in identifying those at need for more counselling and close monitoring [14,15].

The aim of the present cross-sectional study was to assess diet quality and adherence to the dietary guidelines for CF among pediatric patients.

## 2. Materials and Methods

### 2.1. Sample Recruitment

Children and adolescent with CF were recruited from outpatients of the Clinic for Cystic Fibrosis, situated in Aghia Sophia Children’s Hospital, Athens, Greece. Details concerning the recruitment and ethical permission have been previously reported [16,17]. Characteristics of the patients are presented in Table 1.

### 2.2. Anthropometric Indices

Anthropometry was conducted during morning hours, with light clothing and bare feet. Body weight was measured at the nearest 0.1 kg (SECA 874 portable digital scale, Hamburg, Germany) and height to the nearest 0.5 cm (SECA 214 portable stadiometer, Hamburg, Germany), and then body mass index (BMI) was computed. Height-for-age (HAZ) and BMI-for age (BAZ) z-scores were calculated for each patient, based on the Centers for Disease Control (CDC) growth charts [18]. The CDC growth charts were selected on the basis of (i) better comparability with the US and the European Cystic Fibrosis registries, and (ii) our previous observation that the CDC growth charts provide similar estimations of nutritional status compared with the World Health Organization and International Obesity Task Force charts [17,19,20]. According to the BAZ, patients were classified as underweight (BAZ < −2.0), of normal body weight (−2.0 ≤ BAZ < 1.0), overweight or obese (BAZ ≥ 1.0, and BAZ ≥ 2.0, respectively). CF specific classification was used to assess ideal body weight (IBW) attainment (BAZ ≥ 0.0) and possible nutritional failure (BAZ < −1.04) [21]. Children with a HAZ < −2.0 were considered chronically malnourished (stunted).

### 2.3. Dietary Intake, Diet Quality and Adherence to Diet Treatment

Two non-consecutive 24 h dietary recalls were completed for each participant, within a period of 14 calendar days, through interview with an experienced dietician (D.P.) Given that food records are associated with an increased responder burden and higher chances of incorporating changes in the usual eating behavior, we opted for using multiple 24 h dietary recalls [22,23]. For children aged below 9 years old, parental consensus and help in competing the 24 h recalls were sought. Records were then analyzed for total energy intake (TEI), macro- and micro-nutrient intake using the Food Processor software (ESHA, Portland, Oregon).

Diet quality was assessed with the KIDMED questionnaire [24], evaluating adherence to the Mediterranean diet (MD) prototype. The questionnaire is based on 16 questions concerning the frequency of consumption of foods typical to the Mediterranean region, against foods considered as characteristic of the Western diet. The KIDMED is widely used in epidemiology (based on the number of citations), providing reliable estimates of adherence to the MD [25], and has previously been translated and used in the Greek population [26]. Scores < 4 are indicative of low adherence and poor diet quality, scores between 4 and 7 are indicative of mediocre diet quality and scores exceeding 8 are observed among high MD adherers [24].

Dietary intake data were then compared to the Nutrition Guidelines for Cystic Fibrosis in Australia and New Zealand [27], which are considered as the most “complete” CF dietary guidelines in terms of CF-specific recommendations [28]. Participants were categorized according to those adhering and/or exceeding dietary recommendations, or not. The adequate intake of nutrients not included in the aforementioned guidelines was assessed through the relevant population reference intake (PRI) level for each nutrient set by the European Food Safety Authority [29].

### 2.4. Ethical Approval

The parents/guardians of all subjects provided informed consent for inclusion before participating in the study. The study was conducted in accordance with the Declaration of Helsinki, and the protocol was approved by the Ethics Committees of both Aghia Sophia Children’s Hospital, and University of West Attica (reference number 16084/14-07-15).

### 2.5. Statistical Analyses

Data distribution was graphically explored with Q-Q plots. Continuous variables are presented as means ± standard deviation (for normally distributed variables), or medians (1st and 3rd quartile) (for non-normally distributed variables). Categorical variables are presented as relative frequencies and percentages. Differences between continuous variables were assessed with independent t-test (for normally distributed variables), or the Mann–Whitney U test (non-normally distributed variables). Chi-square or the Fisher’s exact test was employed to explore differences between categorical variables. The relatively small number of participants did not allow for the performance of regression analyses.

The level of significance was set at *α* = 0.05. All analyses were carried out on SPSS version 25.0 (IBM, SPSS Inc., Chicago, IL, USA).

## 3. Results

### 3.1. Anthropometric Data

**Figure 1:**
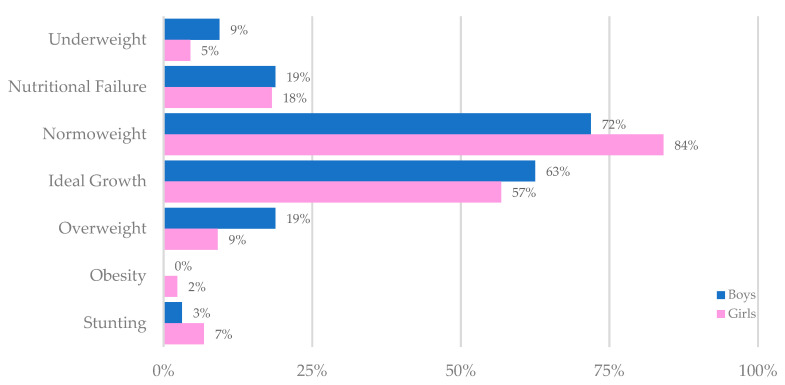
Body weight and height status classification of participants.

In the total sample of girls, and boys, mean values of height and BMI z-scores indicated adequate growth. In particular, for girls, mean BAZ was calculated at 0.02 ± 1.10, and for boys mean BAZ reached 0.01 ± 1.16 (*p* = 0.785 for between sexes comparison). Height z-scores were considered optimal, although mean values were lower than the 50th percentile (girls: −0.19 ± 1.22 and boys: −0.40 ± 0.88, *p* = 0.394). Body weight classification is demonstrated in Figure 1. While nutritional failure was prevalent in 1/5 of both the boys and the girls, the vast majority of the sample attained adequate weight status, with 72% of the boys and 84% of the girls being classified as normoweight, and 60% of participants from both sexes having reached the target CF-specific BMI. Stunting prevailed in few participants. No significant difference was detected in body weight and height status between boys and girls (*p* > 0.05 for all comparisons).

### 3.2. Adequacy in the Dietary Intake

All of the participating children and adolescents (100%) exceeded the estimated energy requirement (EER) intake (Table 2). Protein and fat were adequately consumed by 84.4% and 87.5% of the boys, and 75% and 88.6% of the girls, respectively. On the other hand, carbohydrate and fiber intakes was suboptimal, with only 1/3 of the patients meeting the respective guidance. None of the participants achieved the recommended intake for saturated fatty acids (SFA), with all exceeding the suggested daily consumption levels. Fiber intake was adequate for a mere 40.6% of the boys and 45.5% of the girls, respectively.

With regards to the fat-soluble vitamins’ intake, small proportions of children met the recommended intake for vitamin D and vitamin E, whereas vitamin A intake was adequate for 45.5% of the boys and 60% of the girls. With reference to the mineral intake, adequate consumption was observed by the majority of children concerning all examined minerals, with the exception of boys’ magnesium intake which was suboptimal.

No differences were observed between sexes in the proportion of boys and girls failing within the dietary recommendations, or those exceeding the recommended intakes.

Dietary intake data were also analyzed according to the attainment of IBW and the diagnosis of nutritional failure (data not shown). Children categorized below the 50th percentile of BMI consumed more TEI compared to those exceeding the 50^th^ percentile, both in terms of crude energy intake (2789 ± 728 vs. 2376 ± 642 kcal/day, *p* = 0.014) and expressed as a percentage (%) of the recommended intake (143.8 ± 46.3 and vs. 115.5 ± 38.6%, respectively, *p* = 0.007). Consequently, more children under the 50th BMI percentile consumed a diet within the recommended levels for energy intake (77.4 vs. 53.3%, *p* = 0.032). No other differences were noted for other macro- or micro-nutrients. Children at nutritional failure (BMI < 15th percentile) did not demonstrate any differences in the TEI or nutrient intake as compared to the children with greater BMI. However, a marginal trend was revealed, demonstrating that fewer children with nutritional failure consumed adequate fat (expressed in g) as compared to their heavier counterparts (67.7% vs. 92.9%, *p* = 0.058).

### 3.3. Quality of the Dietary Intake—Mediterranean Diet Adherence

Dietary quality indices are presented in Table 3. The majority of the patients adopted a diet of poor or moderate diet quality (75%), whereas a trend for more boys achieving a mediocre score in the KIDMED, and more girls scoring high in diet quality was revealed. More boys than girls consumed food at fast food restaurants more than once per week (65.7 vs. 37.5%, *p* = 0.014), whereas a trend for more boys having dairy for breakfast than girls was present. The majority of participants scored low in questions regarding the core food groups of the Mediterranean dietary pattern, namely daily fruit and vegetables intake, and the frequency of consumption of fish, pulses and nuts within a week.

Girls scoring low in KIDMED were less likely to report an adequate fiber intake (*p* = 0.006); the reverse was true when girls scored high in KIDMED (*p* = 0.014) (data not shown). Boys scoring high in the KIDMED questionnaire were more likely to consume fat adequately (*p* = 0.043) (data not shown). The KIDMED did not correlate with any dietary factor. No other differences were detected regarding diet quality and nutrient adequacy.

## 4. Discussion

The findings of the present study suggest that Greek school-aged children with CF adhere to a diet adequate in energy, protein and fat intake, that is mostly of low or moderate quality. At the same time, our observations suggest that there is room for improvement in terms of carbohydrate and fiber intake, as well as certain minerals and vitamins.

Relevant to energy intake, all of the examined children were found to consume a diet with an energy content > 110% of the estimated average energy intake for children. These findings are in line with a recent Australian study, indicating that children with CF consumed a diet with a median energy content of 158% of the recommended energy intake [30]. On the other hand, an early report showed that children with CF failed to meet recommendations regarding energy intake [31]. Some more recent data suggest that children with CF marginally met energy recommendations, even though they consumed more energy as compared to healthy controls [32]. These discrepancies may be explained by the different methods of dietary assessment employed in the aforementioned studies. Nevertheless, these differences in between earlier and more recent studies may also be indicative of the vast improvements in dietary counselling in children with CF that have been noted in the past 25 years. At the same time, as indicated by the body weight of participants herein, in their majority they consisted of well-nourished patients, given that for most attainment of IBW and physiological lung function was apparent. According to an Irish study, parents of children with CF often use IBW attainment as a criterion for the children’s health status, influencing their approaches to the dietary management of the disease [33].

With regards to the macronutrient intake, the present sample consumed a diet high in fat, that was also adequate in protein. The latter is known to reduce muscle and bone mineral loss and is associated with improved survival [12]. An early clinical trial revealed that within a short period of supplementation, high protein intake was associated with increased whole-body protein synthesis rates among stunted children with CF [34]. In parallel, a diet high in protein appears to be highly anabolic, even in patients with CF recovering from exacerbations, or those with muscle loss [12]. According to Engelen [12], despite its importance, protein is often under-regarded in the dietary management of children with CF, although that was not the case in our sample.

Adherence to a diet high in fat has been the cornerstone of CF treatment for more than 25 years [35]. In the present analysis, fat intake of most of the participants exceeded the recommended range between 35% and 40% of the daily energy consumption. Previous studies in children with CF have recorded fat intakes within the 30–35% range [31,36,37], or even greater [32,35,38], indicating a unanimous adherence to the fat intake recommendation. Nevertheless, the postulated problem in high-fat diets is the correspondingly elevated SFA content [35,38] which, for more than 50 years, was considered as harmful for cardiovascular health. As a result of their high-fat diet, most children with CF have been reported to exceed the recommendations regarding SFA intake [32,35]. More recently, evidence of higher quality, including meta-analyses, contradicts this narrative, indicating that SFA intake has either positive, or null effect on cardiovascular health based on dose-response analyses [39,40]. On the other hand, children with CF often demonstrate essential fatty acid deficiencies, with some being non-responsive to supplementation treatment due to multiple potential impediments leading to malabsorption issues, indicating that the disease is associated with a cascade of health issues requiring adequacy in the intake of fat [41,42,43]. In parallel, despite the reported high intakes of fat, serum lipid levels are often within normal ranges, even among adults with CF who have adhered to a high-fat diet for a longer period of time [44]. Moreover, research on children has failed to associate fat intake to the lipidemic profile in CF [38], whereas on the other hand, it had been suggested that the high prevalence of hypertriglyceridemia observed in CF might be associated with an increased carbohydrate intake, and not the result of the high-fat diet [45]. Nevertheless, recommendations regarding the intake of SFA in CF retain the former low recommended SFA intake values, despite the existing evidence controversy and scientific debate [46,47].

Participants with CF exhibited a low mean carbohydrate intake. The positives of a low carbohydrate consumption are associated with the reduced sugar and refined cereals intake, whereas an important limitation stems from the often-inadequate fiber content of the diet. As a result, the fiber consumption of the participating children was indeed below recommendations for the majority of participants, with approximately 1/3 of the sample fulfilling the daily intake goal. Previous studies have also highlighted the low fiber intake of children with CF as compared to their healthy peers [48], whereas many children with CF encounter digestive and gastrointestinal issues which might be related to low fiber intake [49]. An older Belgian study failed to associate fiber consumption to gastrointestinal complains and distal intestinal obstruction syndrome (DIOS) among children with CF [50], however, the design was cross-sectional, not allowing for the establishment of a causal relationship. Nevertheless, fiber is a common constituent of ready-to-use supplemental food (RUSF) for children with CF [51], indicating that fiber is regarded as a key nutrient, often consumed inadequately among children with CF.

With reference to micronutrients, mineral intakes have not received adequate attention in CF, in contrast with the fat-soluble vitamins, which are routinely assessed in serum and supplemented orally [52,53]. Our patients exhibited comparable micronutrient intake with an Australian pediatric cohort of CF [54]. While for most micronutrients, the Australian study reported somewhat higher intakes than those we recorded, the largest differences were observed for vitamin A and magnesium intakes. These findings are also in line with findings from the general healthy Greek children, that have reported insufficient intakes of vitamin A and magnesium [55,56].

The MD is a regional dietary pattern with numerous health benefits for the general and disease-specific population [57,58,59,60]. Adherence to the diet had been suggested as a prototype dietary pattern favorable for the management of CF [61]. Although randomized controlled trials and intervention studies on children with CF are yet to be undertaken, the ILERVAS project revealed that adherence to a combination of lifestyle interventions including MD and physical activity was associated with an improved lung function, including higher forced vital capacity (FVC) and forced expired volume (FEV1) in the first second [62]. In the present sample, most participants failed to adhere to the MD pattern, demonstrating low adherence. However, according to the literature, low adherence is also apparent in most children and adolescents inhabiting the Mediterranean region [63,64]. Furthermore, pediatric patients with CF have been known to endorse an energy-dense, yet nutrient-poor diet, that is indicative of a diet low in quality [30]. In the aging population with CF, awareness is raised regarding cardiovascular risk. Low dietary quality has been independently associated with indices of poor cardiovascular health in apparently healthy children [65], whereas poor dietary indices contribute to the burden of disease into adulthood [66]. In light of the aforementioned findings, beyond energy and macronutrient intake, dietary counselling for CF should also advocate for a diet of higher quality, that also targets optimal micronutrient intake.

Limitations of the study include its cross-sectional design and relatively small sample, recruited from one clinic only. Nevertheless, the clinic is the largest in Greece, situated in the capital, Athens, with the largest number of CF pediatric patients. Addition of a control group of healthy children could have allowed for comparisons of our cohort of CF patients with the general population, a lack we faced due to limited resources. Dietary intake was assessed by 2 non-consecutive dietary recalls. Two 24-h dietary recalls are widely used in large populational studies like the National Health and Nutrition Examination Survey (NHANES), alleviate the burden of the patients, the mode of delivery does not affect eating patterns, and are known to be valid methods for the assessment of children’s dietary intake [23,67]. Cross-matching of dietary intake with biochemical data could have provided further insight in the association of dietary intake and health outcomes in CF. On the other hand, the present study consists of the first research item assessing MD in a CF population, adding more data to current knowledge on the nutritional status of children with CF.

## 5. Conclusions

In the majority, the examined children with CF receiving proper nutritional education and advice appear to be well-nourished, adhering to the majority of nutritional recommendations, while failing to meet the goal intakes for fiber and carbohydrate intake. A low adherence to the MD is revealed, indicating that there is still room for further improvement in terms of diet quality, which in turn could also attenuate micronutrient intake.

## Figures and Tables

**Table 1:** Sample characteristics (*n* = 76).

Patient Characteristics	Boys (*n* = 32)	Girls (*n* = 44)	*p* Value
Age (years)	12.4 ± 3.8	11.6 ± 4	0.376
Adolescents (%)	50	31.8	0.109
Body weight (kg)	44.5 ± 18.3	37.4 ± 12.8	0.062
Stature (m)	1.49 ± 0.2	1.41 ± 0.18	0.1
BMI (kg/m^2^)	19.1 ± 3.5	18.2 ± 2.7	0.199
FEV1%	96.7 ± 18.6	99.1 ± 21.9	0.614
Pancreatic Insufficiency (%)	81.3	90.9	0.22
Liver disease (%)	12.5	6.8	0.398
CFRD (%)	3.1	6.8	0.477
Presence of Meconium Ileus (%)	31.3	9.1	0.014
NBS diagnosis (%)	6.3	15.9	0.198
*HomoF508del* (%)	34.4	45.5	0.332
*HeteroF508del* (%)	46.9	40.9	0.604
*P.Aeruginosa* chronic infection (%)	12.5	11.4	0.88
*S.Aureus* chronic infection (%)	0	2.3	0.391

BMI, Body Mass Index; FEV1, Forced expiratory volume in 1 sec; CFRD, Cystic Fibrosis related diabetes; NBS, newborn screening.

**Table 2:** Dietary assessment of girls and boys with cystic fibrosis and compliance with the recommendations *.

	Recorded Daily Dietary Intake				Compliance with the Recommendations		
Energy and Nutrients	Boys (*n* = 32)	Girls (*n* = 44)	*p* Value	Recommendation	Meeting and/or Exceeding Recommendations		
					Boys (%)	Girls (%)	*p* Value
Total Energy intake (kcal/d)	2623 ± 654	2486 ± 738	0.401		-	-	-
TEI (% EER)	122 ± 47	130 ± 42	0.430	110–200% EER	100	100	-
Protein (% TEI)	19.7 ± 7.0	18.0 ± 5.5	0.248	15–20% TEI	84.4	75.0	0.322
Carbohydrate (% TEI)	37.7 ± 8.9	37.9 ± 10.2	0.912	40–65% TEI	37.5	38.6	0.920
Total Fat (g/d)	127 ± 38	123 ± 40	0.677	≥100 g	71.9	72.7	0.935
Total Fat (% TEI)	43.8 ± 7.6	45.1 ± 8.9	0.484	35–40% TEI	87.5	88.6	0.880
Saturated Fat (% TEI)	15.1 ± 3.1	16.5 ± 5.0	0.167	As low as possible	-	-	-
Dietary Fiber (g/d)	13.5 (10.7, 17.2)	13.1 (7.9, 22.9)	0.768	14–30 g	40.6	45.5	0.675
Vitamin A (RE/d)	222 (73, 446)	314 (127, 514)		4–6 yr: 300 RE; 7–10 yrs: 400 RE; 11–14 yr: 600 RE; ≥15 yrs: 750 RE (boys), 650 RE (girls).	45.5	60.0	0.319
Vitamin D (μg/d)	5.7 (3.5, 7.6)	5.7 (3.8, 7.6)		15 μg	4.5	0.0	0.222
Vitamin E (mg/d)	4.5 (6.6, 9.1)	7.8 (4.9, 9.6)		3–9 yrs: 9 mg; ≥10 yrs: 13 mg (boys), 11 mg (girls).	20.5	9.4	0.191
Iron (mg/d)	14.4 (10.6, 19.9)	18.3 (10.2, 29.5)	0.449	4–8 yrs: 10 mg; 9–13 yrs: 8 mg; ≥14 yrs: 11 mg (boys), 15 mg (girls).	71.9	61.4	0.340
Magnesium (mg/d)	237 (181, 329)	246 (207, 289)	0.628	4–8 yrs: 130 mg; 9–13 yrs: 240 mg; ≥14 yrs: 410 mg (boys), 360 mg (girls).	35.5	64.5	0.332
Calcium (mg/d)	1227 (802, 1530)	1271 (895, 1551)	0.474	4–8 yrs: 0.7 g; 9–11 yrs: 1 g; 12–18 yrs: 1.3 g.	71.9	70.5	0.893
Sodium (mg/d) †	2898 (2043, 4080)	2936 (2079, 3524)	0.925	Children: 1–4 g; Adolescents: 6 g.	53.1	63.6	0.357
Zinc (mg/d)	14.8 (7.8, 19.0)	13.1 (9.9, 18.0)	0.958	4–8 yrs: 4 mg; 9–13 yrs: 6 mg; ≥14 yrs: 13 mg (boys), 7 mg (girls).	68.8	79.5	0.283

EER, estimated energy requirements; RE, Retinol equivalents; TEI, Total Energy Intake; yrs, years; * Compared against the Nutrition Guidelines for Cystic Fibrosis in Australia and New Zealand [27]. † refers to sodium naturally occurring in foods.

**Table 3:** Diet quality of children and adolescents with cystic fibrosis according to the KIDMED [24] score.

KIDMED Components and Total Score	Girls (*n* = 44)	Boys (*n* = 32)	*p* Value
Takes a fruit or fruit juice each day (%)	58.3	71.4	0.220
Has a second fruit each day (%)	16.7	17.1	0.954
Has fresh or cooked vegetables regularly once/day (%)	62.5	54.3	0.452
Has fresh or cooked vegetables more than once/day (%)	14.6	20.0	0.515
Consumes fish regularly (at least 2–3/week) (%)	14.6	14.3	0.499
Goes > 1/week to a fast food restaurant (hamburger) (%)	37.5	65.7	0.014
Likes pulses and eats them > 1/week (%)	41.7	40.0	0.673
Consumes pasta or rice almost every day (≥5/week) (%)	100	97.1	0.239
Has cereals or grains (bread, etc.) for breakfast (%)	47.9	68.6	0.061
Consumes nuts regularly (at least 2–3/week) (%)	39.6	28.6	0.299
Uses olive oil at home (%)	97.9	94.3	0.381
Skips breakfast (%)	20.8	20.0	0.685
Has a dairy product for breakfast (yoghurt, milk, etc.) (%)	81.3	94.3	0.084
Has commercially baked goods or pastries for breakfast (%)	10.4	20.0	0.220
Takes two yoghurts and/or some cheese (40 g) daily (%)	93.8	88.6	0.402
Takes sweets and candy several times every day (%)	35.4	22.9	0.187
KIDMED score	5.7 ± 2.6	5.6 ± 2.3	0.860
Low * KIDMED score (%)	25.0	22.9	0.822
Moderate * KIDMED score (%)	43.8	62.9	0.085
High * KIDMED score (%)	31.3	14.3	0.074

* Low KIDMED score was defined as <4, scores between 4 and 7 were considered as indicative of moderate diet quality and scores exceeding >8 were used to identify high Mediterranean diet adherers [24].
